# Case report: A novel *TLK2* variant with a neuropsychiatric phenotype from a Chinese family

**DOI:** 10.3389/fgene.2024.1419027

**Published:** 2024-09-04

**Authors:** Hongmei Huang, Yue Qian, Chenlu Yang, Shijie Li

**Affiliations:** Beijing Children’s Hospital, Capital Medical University, National Center for Children’s Health, Beijing, China

**Keywords:** *TLK2* gene, behavioral disorder, neurodevelopmental disorder, case report, heterozygous variant

## Abstract

**Background:**

Tousled-like kinase 2 (TLK2) gene variant-related neurodevelopmental disorder was recently described. The haploinsufficiency of TLK2 was considered the most likely underlying disease mechanism, leading to a consistent neurodevelopmental phenotype. So far, only four studies, conducted on 49 patients from North America and Europe, have been reported.

**Case presentation:**

In this study, we reported a Chinese family with a *TLK2*-related neuropsychiatric phenotype. The proband, a boy aged 2 years and 6 months, presented with temper tantrums, mood lability, aggressiveness, congenital astigmatism, and distinctive facial dysmorphism. Whole-exome sequencing identified a novel heterozygous variation in *TLK2* gene (c.49dupG, p. E17Gfs*10) in them. His father carried the same *TLK2* gene variant and exhibited anxiety and irritability. The parental grandparents and other family members had no such variation. Moreover, the proband was found to have global developmental delay, autism-like symptoms, and mild elevated homo-vanillic acid (HVA) and 2,3-dihydroxy-2-methylbutyric acid levels tested in urine.

**Conclusion:**

Herein, we identified a novel *TLK2* variant from a Chinese family and reported a new neuropsychiatric phenotype. This study also expanded the genotype profile of the newly defined *TLK2*-related neurodevelopmental disorder.

## Introduction

Neurodevelopmental disorders (NDDs) are genetically heterogeneous diseases. Studies have shown that the genetic etiology of neurodevelopmental disorders can be found in up to 47% of patients with NDD (Woods et al., 2022). Tousled-like kinase 2 (*TLK2*) is located at 17q23.2, contains 22 exons, and encodes a ubiquitous 717-aa protein, which plays an important role in DNA replication, cell cycle checkpoint, recovery, and chromatin remodeling (Mortuza et al., 2018). The *TLK2* gene variant was first described to be a candidate gene of NDDs in a cohort study in 2016 (Lelieveld et al., 2016). Following the initial publication, four additional reports were published, associating this gene with neurodevelopmental disorder (Reijnders et al., 2018; Topf et al., 2020; Pavinato et al., 2022; Woods et al., 2022). One study described a patient with a homozygous *TLK2* variant, leading to an autosomal recessive severe neurodevelopmental disorder (Topf et al., 2020). Reijnders et al. identified a large number of individuals (38 probands and 2 affected mothers) with heterozygous mutations in *TLK2* gene showed similar clinical characteristics, including mild-to-moderate intellectual developmental disorder, behavioral problems, gastrointestinal disorders, and typical facial dysmorphism (Reijnders et al., 2018). Recently, another two studies reported pathogenic *TLK2* variants in eight patients with global developmental delay and also described that some novel phenotypes include sleep disturbance, non-differentiation of lateral semi-circular canals, vesico-ureteric reflux, and bilateral periauricular skin tags (Woods et al., 2022).

Totally, 39 *TLK2* gene variants in 49 patients were reported to exhibit *TLK2*-related neurodevelopmental disorders in four studies. All cases were identified from North America and Europe, and more than two-thirds of them, from two countries, namely, the Netherlands and the UK (1, 4-6). With an estimated prevalence of 1/566 (17/9,625) of *TLK2* variants in probands recruited to the NDD cohort study, it is expected that a larger number of individuals with *TLK2* variants is present worldwide (Reijnders et al., 2018).

Here, we presented a Chinese family with *TLK2*-related neurodevelopmental disorder. A novel heterozygous variant (c.49dupG, p. E17Gfs*10) in *TLK2* gene, was identified in a 2-year-old boy with mild global developmental delay, autism-like symptoms, and moderate-to-severe behavior problems. His father also carries this *TLK2* variant and presented temper tantrums, mood lability, and aggressiveness. They also have congenital astigmatism and distinctive facial dysmorphism. The boy’s grandparents and other family members have no such variation.

### Case presentation

The proband was a boy aged 2 years and 6 months with behavioral problems. He was recommended to our Pediatric Developmental Behavior Clinic because he presented temper tantrums, mood lability, self-injurious behavior, and aggressiveness toward others at home. He was full-term born to nonconsanguineous parents by selective C-section delivery, G1P1, with birth weight 3300 g (75–90th percentile), and birth height 50.0 cm (50–75th percentile). There were no other medical issues during pregnancy. It was observed that he was able to sit alone at 6 months, to walk alone at 13 months, and to speak the first meaningful words after 2 years of age.

Physical examination revealed his height was 95 cm (within the 50 to 75 centiles for his age), weight was 15 kg (within the 75 to 90 centiles for his age), and head circumference was 48.5 cm (within the 25 to 50 centiles for his age). Distinctive facial features were noticed including low-setting ear, flat nasal bridge with a broad nasal tip, and downturned mouth. The Gesell developmental diagnosis scale was used to assess the proband’s development quotient (DQ): adaptability DQ 46, gross motor DQ 64, fine motor DQ 47, sociality DQ 51, and language DQ 60. The result of the S-M social living ability test score was 7. Positive results were found in the Clancy Behavior Scale (CBS) and Modified Checklist for Autism in Toddlers (M-CHAT). The combined communication and social interaction score was 17 according to the Autism Diagnostic Observation Schedule (ADOS), which exceeded ADOS cutoff scores for autism spectrum disorder (ASD).

He exhibited poor communication and social interactions in an unfamiliar environment, as well as autism-like behavioral traits, hand-flapping movement, echolalia, and repetitive language. In addition, he also presented irritability, mood lability, and hyperactive behavior in the behavior observation room. Global developmental delay and ASD were considered by clinical professionals in accordance with the DSM-5 and clinical manifestations.

The proband showed elevated homo-vanillic acid and 2,3-dihydroxy-2-methylbutyric acid levels in urine. Other biochemical parameters were within the normal range, including the serum levels of neuron-specific enolase, lactic acid, cysteine, very long-chain fatty acids, amino acids, blood lipids, cholesterol, and hormones including TSH, FT4, LH, and FSH. No significant abnormalities were detected via EEG and head MRI. Congenital hyperopia in the right eye was found during the eye examination. There was no abnormal finding in hearing examination, echocardiography, abdominal ultrasound, and gastrointestinal ultrasound.

Furthermore, the proband’s father presented temper tantrums and mood lability, which affected his work and family to some extent. He reported marginal learning difficulty in his childhood but could not remember the developmental history in detail. He also presented distinctive facial features, prominent nasal bridge, and a broad nasal tip. The mother of the proband showed normal physical growth and mental development. No psychiatric or physical disorders were reported in maternal and paternal families.

**FIGURE 1:**
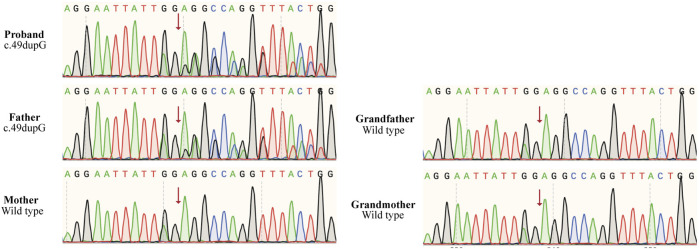
Sanger sequencing chromatograms of mutation c.49dupG (p.E17Gfs*10) in *TLK2* gene, and the position is indicated by an arrow. The proband and his father were heterozygous of the mutated gene, while his mother and grandparents were normal.

**FIGURE 2:**
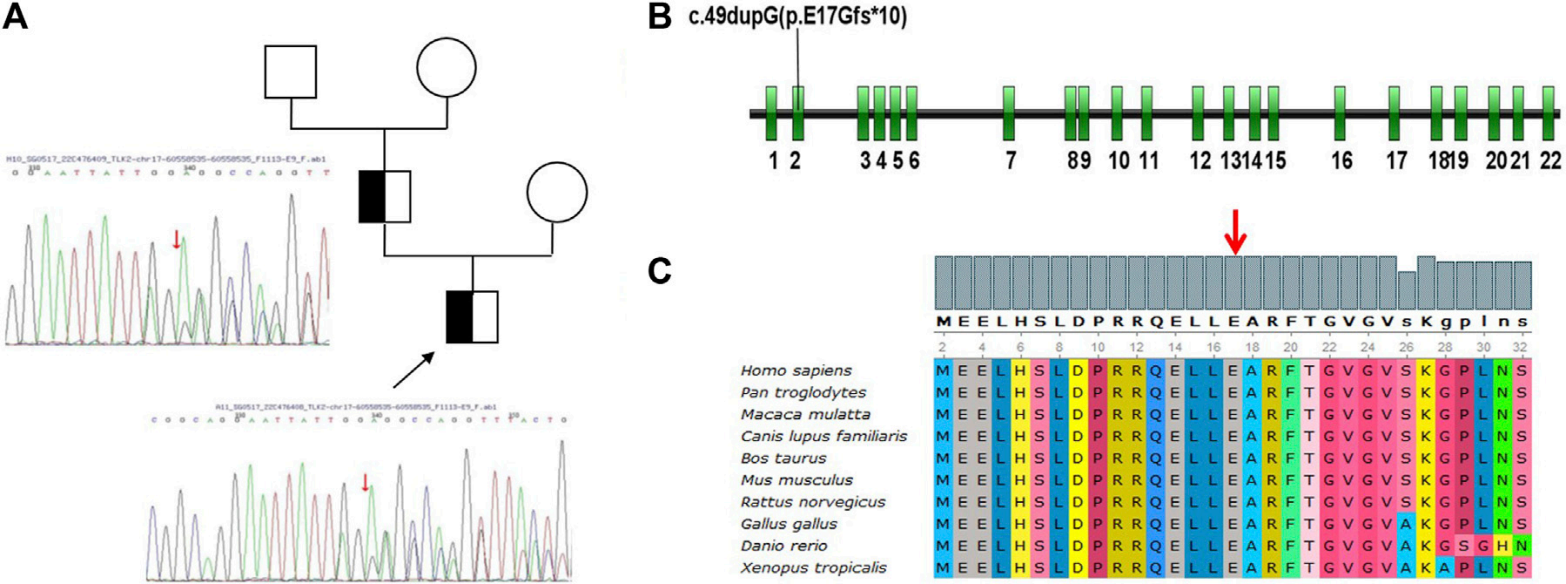
**(A)** Pedigrees of the two individuals issued from the Chinese families. The black arrow indicates the proband. The affected individuals with the homozygous *TLK2* variant were indicated with a half-black square. Empty circles or squares indicated other non-carriers. **(B)** Exon models of *TLK2* gene showing the location of the variant (c.49dupG, p. E17Gfs*10) in Exon 2. **(C)** Multi-species conservation alignment of TLK2 homologs. The red arrow indicates the site of the variant.

Cytogenetics showed a normal male karyotype (46, XY), testing for Fragile X, and Leigh syndrome and other mitochondrial diseases were negative; microarray analysis detected no clinically significant imbalance. We performed whole-exome sequencing and the copy number variation test. A novel frameshift variant (c.49dupG, p. E17Gfs*10) was identified in the *TLK2* gene of the proband, which may cause loss of gene function, and his father was also found to carry this *de novo* variant (Figure 1). However, the grandparents and mother of this proband did not have the variant (Figure 1). According to ACMG guidelines, the variation is preliminarily determined as likely pathogenic variation (PVS1 + PM2) (Richards et al., 2015). This mutation had not been reported in the literature and ClinVar database. (Figure 2).

## Discussion and conclusion

We reported a Chinese family with a novel heterozygous variant (c.49dupG, p. E17Gfs*10) in *TLK2* gene. The proband, a 2-year-old boy, inherited this TLK2 variant from his father who carried the *de novo* TLK2 variant. These two affected individuals both presented prominent behavioral problems like temper tantrums, mood lability, and aggressiveness. Similar distinctive facial features, including an abnormal nasal bridge, broad nasal tip, and thin vermilion of the upper lip, were observed in them. The proband also displayed global developmental delay and autism-like symptoms, consistent with *TLK2*-related neurodevelopmental disorder in the available clinical literature (Woods et al., 2022). In addition, we observed mild elevated homo-vanillic acid (HVA) and 2,3-dihydroxy-2-methylbutyric acid levels in the urine of the proband. A high HVA level in urine, first reported in the affected individual with the *TLK2* variant, may relate to the prominent behavioral problems in the proband.

**FIGURE 3:**
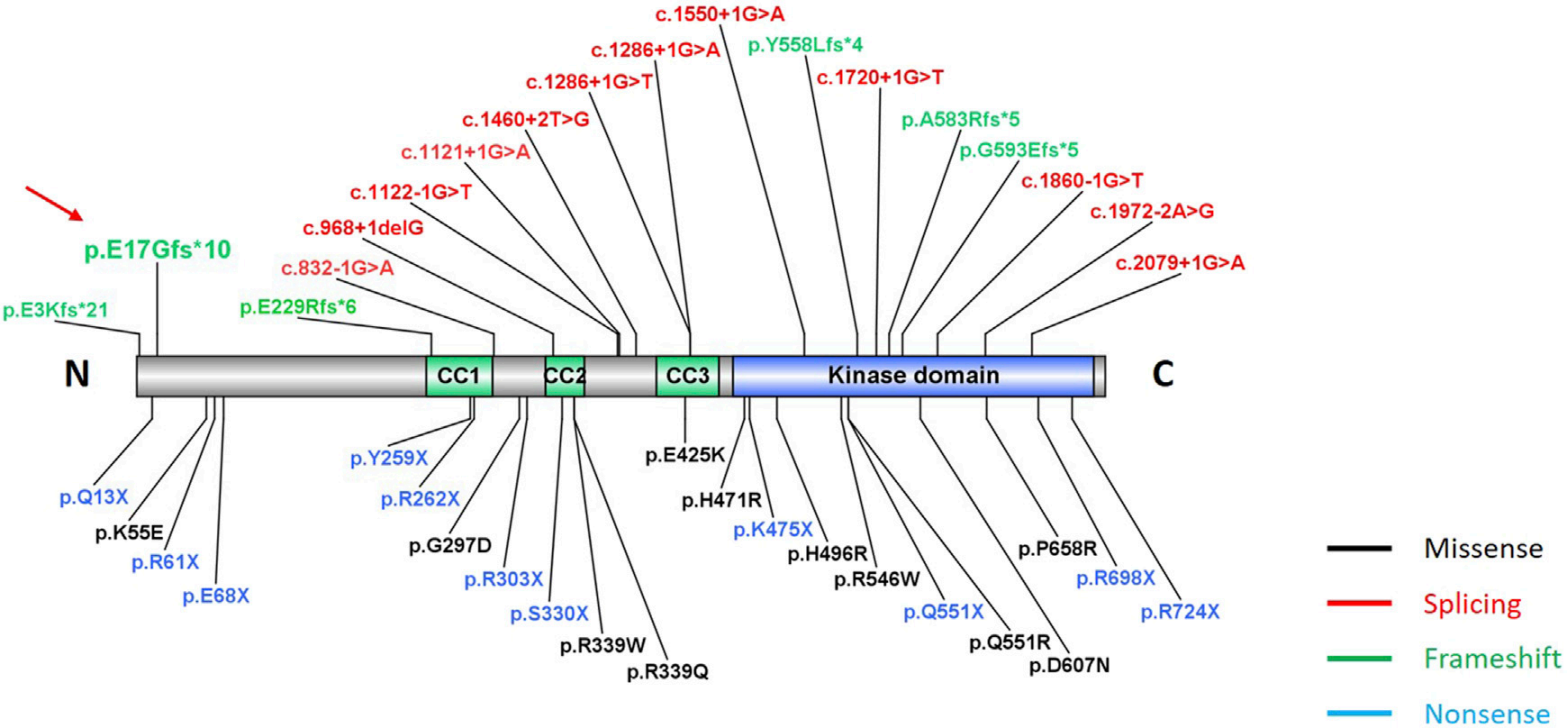
Location of pathogenic variants in *TLK2* gene. Totally, 40 variants in our study and previous literature studies are showed here, including missense (black), splicing (red), frameshift (green), and nonsense (blue) variants. The red arrow indicates the novel frameshift variant in our study. The three green functional domains are coiled-coil domains (CC1, CC2, and CC3), and the blue domain is the kinase domain.

This novel *TLK2* variant (c.49dupG, p. E17Gfs*10) was predicted to result in nonsense-mediated mRNA decay, consistent with a loss of function and haploinsufficiency (Lambert et al., 2020). It was a novel variant that had not been reported before (Figure 3). It has been estimated that approximately 30% of inherited human diseases are due to the presence of premature termination codons or frameshifts that induce nonsense codons in mRNAs (Popp and Maquat, 2016). In the majority of reported cases, the disease is likely due to TLK2 haploinsufficiency as most of cases are heterozygous for loss-of-function alleles, which is in line with the strong constraint against loss-of-function variants in the gene (pLI = 1, GnomAD database) (Pavinato et al., 2022). The site was highly conserved in different species, which supported that the frameshift mutation within the N-terminal domain was a candidate for disease causation. TLK2 polypeptide is a predominantly nuclear protein composed of an N-terminal region harboring a nuclear localization signal, a middle region of helices predicted to contain three coiled coils (CC), and a C-terminal kinase domain (Mortuza et al., 2018). The N-terminal domain of TLK2 protein contains a nuclear localization signal, and mutants lacking the first 160 amino acids resulted in failing to localize to the nucleus (Mortuza et al., 2018). TLK2 activation requires dimerization through an N-terminal coiled coil motif, suggesting that inactive mutants could have a dominant negative effect (Mortuza et al., 2018). Thus, the predominant pathogenic mechanism of *TLK2* mutations appears to be a reduction in its overall activity. Moreover, *TLK2* variants were indicated to alter proximal interactions with many genes encoding proteins involved in chromatin remodeling that are associated with neurodevelopmental disorders (Pavinato et al., 2022).

In addition, we identified, for the first time, HVA and 2,3-dihydroxy-2-methylbutyric acid levels were increased in the urine of the proband with *TLK2* variants. It was not clear whether the high HVA and 2,3-dihydroxy-2-methylbutyric acid levels were involved in *TLK2*-related neurodevelopmental disorder. This might be one of causes for the severe behavioral disorder in the proband. HVA is the last metabolite of the neurotransmitter dopamine, and 2,3-dihydroxy-3-methylpentanoic acid is an intermediate metabolite of isoleucine in the human body (Gatarek and Kaluzna-Czaplinska, 2022). Isoleucine, a branched-chain amino acid, is an essential amino acid involved in biological functions of brain development and recently linked with autism (Anand et al., 2021). In addition, the elevated HVA level in urine may be resulted from the reduced dopamine β-hydroxylase enzyme activity, which is a very important enzyme in catecholamine metabolism and converts dopamine to norepinephrine in noradrenergic neurons and adrenergic neurons (Gevi et al., 2020; Gatarek and Kaluzna-Czaplinska, 2022). High levels of HVA indicate that oxidative stress caused by superoxide free radicals affects the sympathetic nervous system (Shaw, 2010; Fu et al., 2020). In the literature, a relationship between the severity of ASD symptoms and HVA levels has been observed. High HVA levels in the urine of children with ASD suggest some dopaminergic system disturbance, causing mood disorders, disorders of social relationships, aggression, and also repeated behaviors (Kaluzna-Czaplinska et al., 2010).

In summary, we described a Chinese family with a novel heterozygous *TLK2* variant, leading to behavior disorders, global developmental delay, and typical facial dysmorphism. Our finding expanded the genotype of *TLK2* variation in neurodevelopmental disorders and also reinforced the focus on the behavior problems in individuals with *TLK2* variants. Extensive further studies are needed to explore the pathogenesis of variants in *TLK2* gene.

## Data Availability

The data presented in the study are deposited in the China National Genebank (CNGB, https://db.cngb.org/cnsa/), accession number CNP0006065.

## Abbreviations

TLK2, Tousled-like kinase 2; HVA, homo-vanillic acid; NDDs, neurodevelopmental disorders; DQ, development quotient; TSH, thyroid-stimulating hormone; FT4, free thyroxine; LH, luteinizing hormone; FSH: follicle-stimulating hormone; EEG, electroencephalogram; MRI, magnetic resonance imaging; CBS, Clancy Behavior Scale; M-CHAT, Modified Checklist for Autism in Toddlers; ASD, autism spectrum disorder; CC, coiled coil.
